# 692. *Coccidioides sp.* Infective Endocarditis: A Review of the Literature

**DOI:** 10.1093/ofid/ofab466.889

**Published:** 2021-12-04

**Authors:** Sabirah N Kasule, Michael Apolinario, Christopher Saling, Janis E Blair, Lisa Speiser, Holenarasipur R Vikram

**Affiliations:** 1 Mayo Clinic in Arizona, Phoenix, Arizona; 2 Mayo Clinic Hospital, Phoenix, Arizona

## Abstract

**Background:**

Despite the endemic nature of *Coccidioides* sp. to the American Southwest, the incidence *Coccidioides sp.* infective endocarditis (CIE) is rare. Following successful treatment of a patient with CIE at our institution, we reviewed the literature to identify trends in disease presentation, patient characteristics, and outcomes.

**Methods:**

We reviewed all cases of CIE reported since 1938. Details including patient demographics, underlying immunodeficiency, time to diagnosis, treatment, and outcome were collected for analysis of diagnostic challenges and survival.

**Results:**

Including ours, we identified 11 published cases of CIE. The majority (7) occurred in men. 5 patients were of either African American or Hispanic descent. Of the 10 patients with reported ages, the median age was 35.5 years (range 3 weeks – 61 years). 5 patients had a previous diagnosis of coccidioidomycosis and only 3 had an immunocompromising condition. These comprised pregnancy, heart transplant, and juvenile inflammatory arthritis. Three cases had multi-valvular involvement, but the majority affected the mitral (5) and the aortic (4) valves. Only 2 of the 11 cases involved a prosthetic valve. Of the 8 cases with reported blood cultures, only 2 were positive. Ten of the 11 cases had extra-cardiac disease. Complement fixation (CF) titers were heterogenous with a median of 1:32 and a range of 1:1 to 1:2048. There was no obvious correlation between a patient’s CF titer and their survival. Average time to diagnosis was 3.5 months (range 2.5 – 36 months). Diagnosis was made post-mortem in 4 of the 11 cases. 6 patients (54%) did not survive. Notably, 2 of the fatal cases preceded the discovery of amphotericin B (1969) and 4 occurred prior to the discovery of fluconazole (1990). Of the five patients that survived, four required surgical intervention in addition to azole therapy.

**Conclusion:**

CIE is a diagnostic and therapeutic challenge. The diagnosis itself is rare, culture incubation times are long, and the symptoms are often non-specific thus delaying definitive therapy. The introduction of azole therapy appears to have had significant impact on rates of survival. Despite this, successful management of CIE still requires concurrent surgical intervention with aggressive, indefinite anti-fungal therapy.

**Disclosures:**

**All Authors**: No reported disclosures

